# A phase II study of weekly irinotecan in patients with locally advanced or metastatic HER2- negative breast cancer and increased copy numbers of the topoisomerase 1 (*TOP1*) gene: a study protocol

**DOI:** 10.1186/s12885-015-1072-9

**Published:** 2015-02-21

**Authors:** Iben Kümler, Eva Balslev, Jan Stenvang, Nils Brünner, Dorte Nielsen

**Affiliations:** 1 Department of Oncology, Herlev University Hospital, Copenhagen, Denmark; 2 Department of Pathology, Herlev University Hospital, Copenhagen, Denmark; 3 Department of Veterinary Disease Biology, University of Copenhagen, Copenhagen, Denmark

## Abstract

**Background:**

About 20% of patients with primary breast cancer develop metastatic disease during the course of the disease. At this point the disease is considered incurable and thus treatment is aimed at palliation and life prolongation. As many patients will have received both an anthracycline and a taxane in the adjuvant setting, treatment options for metastatic breast cancer are limited. Furthermore response rates for the most commonly used drugs range from around 30% to 12% . Thus new treatment options are needed and preferably coupled to biomarkers predictive of response. Irinotecan is a topoisomerase 1 inhibitor used for decades for the treatment of colorectal cancer. Four studies have investigated the efficacy of irinotecan monotherapy in breast cancer and all have included non-biomarker selected patients. In these studies response rates for irinotecan ranged from 5%-23% and are thus comparable to response rates obtained with drugs commonly used in the metastatic setting. If a predictive biomarker could be identified for irinotecan, response rates might be even higher.

**Methods/Design:**

This multi-centre phase II single arm trial was designed to investigate if patients with metastatic breast cancer and increased expression of the topoisomerase 1 gene have a high likelihood of obtaining a clinical benefit from treatment with irinotecan. Trial recruitment is two-staged as 19 patients are planned to participate in the first part. If less than 7 patients have clinical benefit the trial stops, if more than 7 patients have clinical benefit a total of 40 patients will be included.

**Discussion:**

This ongoing trial is the first to prospectively test copy number of the topoisomerase I gene as a predictive biomarker of response to irinotecan.

**Trial registration:**

EudraCT number 2012-002348-26.

## Background

Breast cancer (BC) is the most common malignancy among women in the western world. Despite improvements in the adjuvant treatment around 20% of patients with primary BC will develop recurrence [1]. Metastatic breast cancer (mBC) is considered incurable in the vast majority of cases and thus treatment is aimed at palliation and life prolongation. Depending on estrogen (ER) and human epidermal receptor 2 (HER2) status, treatment options include a handful of cytostatic drugs among which the anthracyclines and the taxanes are the most efficient. Given as first-line treatment to chemo-naïve patients, anthracyclines have shown response rates (RR) of up to 70% [2,3]. Among patients exposed to adjuvant non-anthracycline containing chemotherapy RRs are around 50% [2,4,5], while these figures drop to around 36%-38% when given to patiets previopusly treated with anthracyclines in the adjuvant setting [6]. Taxanes have shown RRs of approximately 40% in the first-line setting [7,8], dropping to 26-30% as second-line treatment [9]. The proportion of patients with primary BC receiving some kind of adjuvant therapy has increased during the past 20 years. Although not all patients receive adjuvant chemotherapy still a growing number of patients will have been exposed to both anthracyclines and taxanes at the time of recurrence. In these cases or following relapse on either treatment capecitabine or vinorelbine will often be considered as rational treatment options. Although no specific recommendations are given in international guidelines regarding the choice of drug for second- or third line treatment, unanimously guidelines recommend sequential monotherapy [10,11]. Capecitabine has shown RRs in the range 20% to 30% as first-line treatment [12-15] and vinorelbine has showed RRs of 15%-25% when given as second- or third line treatment [16-18]. Eribulin a new microtubule inhibitor has shown RRs of 12% in heavily pre-treated patients whereas the old drug gemcitabine is effective in 20% to 37% of patients depending on line of treatment [19-22]. From these figures it is evident that the majority of patients are deriving no benefit from treatment with these agents but nevertheless suffer the associated side effects. New individualized treatment options are needed for these patients. In order to improve the therapeutic index, preferably all drugs should be coupled to biomarkers predictable of response to the treatment. This is already in use with anti-ER and anti-HER2 treatments but for the cytotoxic chemotherapeutics no such biomarkers are developed to a stage where they can be implemented in daily clinical breast cancer management.

### Irinotecan

Irinotecan is a topoisomerase 1 (Top 1) inhibitor acting as a pro-drug and is enzymatically converted to the active metabolite SN-38 [23]. Like other Top 1 inhibitors it exerts its mechanism of action through binding to the Top 1-DNA complex during replication and transcription and thereby maintaining single-strand DNA breaks, ultimately resulting in the formation of double-stranded DNA breaks, which causes cell death [24]. Irinotecan has been used in the treatment of colorectal cancer (CRC) for decades. Several studies have tried to establish a correlation between expression of the *TOP1* gene or protein and response to irinotecan. Results have been conflicting and study methods have varied significantly making comparisons of the various studies quite difficult. Today no standardized methods for detecting Top 1 protein expression have been established and no validated scoring systems exists for neither gene nor protein expressions. Nevertheless, some studies have found a correlation between *TOP1* gene as well as protein expressions and response to irinotecan [25,26]. From our laboratory, we have published a significant correlation between *TOP1* gene copy number and sensitivity to SN38 in 10 human CRC cell lines [27] and very recently we published on the association between *TOP1* gene copy number and benefit from irinotecan treatment in metastatic CRC [28]. These studies suggest that *TOP1* gene copy number determinations can be used as predictive biomarker for irinotecan treatment in CRC patients. However, additional clinical studies are need to establish *TOP1* gene copy number as an irinotecan predictive biomarker in CRC patients and further as a predictive biomarker for irinotecan treatment of BC patients.

### Irinotecan in BC

Few studies on irinotecan in BC have been published. A systematic review identified 19 trials [29]. No phase III or randomized phase II trials have been published except for trials randomizing between different dosing schedules of irinotecan monotherapy [30,31] and only one study included more than 100 patients [30]. All trials included unselected patients and prior treatment with an anthracycline and/or a taxane was required in all but one study. Four studies investigated irinotecan as monotherapy. Treatment schedules varied among the studies as three studies investigated intravenous irinotecan given either weekly for 4 weeks followed by two weeks break, given every other week or given every third week [30,32,33]. The last study investigated oral irinotecan [31]. RRs ranged from 5% to 23% with the higher response rate found in a study by Perez et al. in which two different schedules of irinotecan were compared [29]. One hundred and three women were included and a maximum of two prior treatment regimens for mBC was allowed. Patients received either weekly irinotecan for four weeks followed by two weeks break (q 42d) or irinotecan every 3 weeks (q 21d). RR in the q 42d arm was 23% (CI 13%-37%) compared to 14% (CI 6-26%) in the q 21d arm. Median response duration was 4.9 months for patients in the q 42d arm compared to 4.2 in the q 21d arm and median OS was 9.7 months (CI 8.0-14.2 months) and 8.6 (CI 7.0-12.3 months), respectively [30]. The two remaining studies on intravenously irinotecan both showed RR of approximately 6% [32,33]. However, one study was a retrospective study of 20 patients previously treated with both an anthracycline and docetaxel [33] while the other study included 18 patients of which 72% had received three or more chemotherapy regimens for metastatic disease [32]. The last study was on oral irinotecan [31]. A more recent study investigated the new polymer conjugate of irinotecan; etirinotecan (NKTR-102). A total of 70 patients were included with a maximum of two prior treatment regimens for metastatic disease. Etirinotecan was administered every 14 days (q14d) or every 21 days (q21d). Progression free survival (PFS) was 3.5 months in the q14d arm and 5.3 months in the q21d arm and overall survival (OS) was 8.8 months and 13.1 months, respectively. However, the RR of 32% was superior in the q14d arm compared to the RR of 26% in the q21d arm [34]. Thus RRs for irinotecan are comparable to those found for taxanes used in second-line and to vinorelbine, gemcitabine and eriblulin used in second- or later lines of treatment. However, as all of the previously conducted studies with irinotecan in BC have included unselected patients, the RR for irinotecan could possibly be higher if a biomarker for response could be identified.

### Topoisomerase 1 in BC

In order to make TOP1 a clinically relevant biomarker for response to irinotecan, overexpression of the protein would have to occur in a fairly large part of patients. Only one published study has investigated the frequency of Top 1 protein expression in BC. A small study including samples from 22 primary BC reported increased expression of Top 1 by immunohistochemistry (IHC) in 41% of the investigated samples indicating that this aberration is quite common in BC [35].

Conclusively, the available data on irinotecan for the treatment of mBC are very scant and insufficient but they all point to a potential role of this drug in treatment of mBC patient with prior taxane and anthracycline treatment. An objective RR to irinotecan treatment in such patients appears to be approximately 25-30% and therefore calls for identification of irinotecan predictive biomarkers to avoid unnecessary drug induced site effects in non-responsive patients.

The aim of the below presented clinical study is to investigate if patients with mBC and increased gene expression of *TOP 1* in their cancer cells have a high likelihood of obtaining a clinical benefit when treated with irinotecan.

## Methods/Design

### Design

This study is a prospective non-randomized phase II trial of weekly irinotecan in patients with locally advanced or metastatic HER2- negative BC and increased copy numbers of the *TOP1* gene in their cancer cells.

#### Participants

The study is a multi-centre trial including 7 oncologic centres in Denmark. To be eligible for inclusion patients must provide written informed consent before any study related procedures. All patients must be above the age of 18, have performance status 0–2 according to the Eastern Cooperative Oncology Group (ECOG) and a life expectancy of ≥ 3 months. Furthermore, patients should have HER2-negative mBC and measurable disease according to RECIST 1.1 and with a maximum of 4 prior chemotherapy regimens for metastatic disease; all previous endocrine treatment is allowed. A neutrophil count of ≥ 1.5 x 10^9^ and platelets ≥ 100 x 10^9^ are required as well as adequate liver function. Finally, patients should have increased copy number of the *TOP1* gene in either their primary or metastatic lesion defined as ≥ 4 gene copy numbers or a *TOP1*/CEN-20 ratio of ≥2.

Exclusion criteria include current or previous other malignant diseases except basal cell carcinoma of the skin and carcinoma in situ cervices uteri, cytotoxic or experimental therapy within 14 days before enrolment and evidence of active CNS metastases. Furthermore, patients who are pregnant, breastfeeding or of childbearing potential and not using adequate non-hormonal contraception will be excluded as will patients with active infections or other severe concomitant medical conditions that may hinder the patient’s opportunity of receiving treatment according to the protocol.

#### Treatment

Patients are treated with irinotecan 75 mg/m^2^ weekly for three weeks followed by two weeks break. CT scans are performed prior to initiation of treatment and then every 6 weeks. Treatment is continued until progression or unacceptable toxicity. Toxicity is assessed prior to every treatment according to the common terminology criteria for adverse events (CTCAE, version 4.0).

#### Ethics

The trial was approved by the Ethical Committee of Region Hovedstaden (H-1-2012-066) as well as by the Danish Medical Authority (EudraCT number 2012-002348-26) and the Danish Data Protection Agency (2007-58-0015 /HEH.750.24-64).

#### Study objectives

The primary end point of this study is clinical benefit rate (CBR) defined as the fraction of patients receiving clinical benefit (CB) in the form of complete or partial response or stable disease ≥ 4 months according to RECIST 1.1. Secondary endpoints include PFS, OS and toxicity.

#### Statistics

Number of patients in the study is based on Simon’s two-stage minimax design. Intention-to-treat analysis will be performed. By using a significance level of 0.05 (a = 0.05) and a power of 80% (b = 0.20) 19 patients should be included in the first step in order to find a CBR of at least 30%. Further inclusion ceases if less than 7/19 patients show CB. If 7 or more show CB another 20 patients will be included. If more than a total of 16 patients achieve CB the hypothesis is satisfied.

PFS and OS will be estimated using the Kaplan-Meier method and compared with a log-rank test. Categorical variables are indicated by a median followed by the span. A significance level of 5% will be used. PFS and OS will be assessed in the evaluable population.

### Methods

#### TOP1 gene copy assessment

Prior to inclusion tissue samples from primary and/or metastatic lesions are investigated for *TOP1* and CEN20 gene copy numbers. Whenever possible, tissue microarrays (TMA) are constructed, using Advanced Tissue Arrayer, ATA-100 (Chemicon International, Temecula, CA, USA). Four 1 mm cores cut at 3 μm are obtained from each biopsy. Samples containing small amounts of tumor tissue or otherwise not suited for TMA construction have full sections made. FISH using a *TOP1*/Centromere-20 (CEN-20) probe [27,36] is used to evaluate the *TOP1*/CEN-20 ratio as well as the absolute number of *TOP1* gene copies per cell.

A total of 60 signals are counted per tissue sample. If a *TOP1*/CEN-20 ratio ≥2 or an absolute gene copy number of ≥4 is found the patient is eligible for inclusion.

## Discussion

Facing patients with mBC, clinicians today possess a limited number of treatment options. As many patients will have received both an anthracycline and a taxane during adjuvant treatment only four or five drug options remain. RRs for these, range from approximately 35% to as low as 12% depending on line of treatment. New and non-cross resistant treatment options are required and importantly biomarkers predictive of response to treatments are desperately needed. Despite intensive research the identification of such biomarkers has failed so far. Attempts have been made and earlier studies seemed to indicate that amplification and possible deletion of the *TOP2a* gene, the target of anthracyclines was predictive of response to epirubicin in the adjuvant setting. However, a recent review concluded that results are conflicting and the role of *TOP2a* as a biomarker for response to anthracyclines is still debated [37]. Irinotecan has shown RRs at least comparable to other drugs when given as second- or third-line treatment to unselected patients with mBC. If a suitable biomarker for response could be identified RRs could possible increase and thereby increase the therapeutic index of irinotecan treatment.

Based on published data from CRC patients and CRC cell lines [25-27] we hypothesize that increased copy number of the *TOP1* gene is predictive of efficacy to irinotecan in mBC patients. As no studies have shown a direct relation between *TOP1* gene number and protein expression in BC this hypothesis should ideally be tested by assessing protein expression using IHC. Unfortunately, suitable and validated antibodies as well as standardized protocols for quantification of Top1 IHC are lacking and moreover, a recent publication has emphasized some of the problems related to the use of commercially available TOP1 antibodies for IHC [38]. Thus, we decided to use *TOP1* FISH as a proxy for Top1 IHC. *TOP1* FISH allows for a direct evaluation of gene copy numbers in individual cells. Furthermore, FISH is widely used and a well-established technique in most Pathology Departments.

No prior studies have prospectively used *TOP1* gene copy numbers to predict response to irinotecan and so the threshold for likelihood of response is unknown. In need of specific *TOP1* gene copy number recommendations the cut-off point of either increased copy number defined as ≥ 4 gene copy numbers or a *TOP1*/CEN-20 ratio of ≥2 was chosen.

The aim of our clinical study is to take a further step towards individualized mBC treatment. If irinotecan is effective in patients with increased *TOP1* gene copy number this would lead to a novel individualized treatment option for a sup-group of patients with mBC and it will also spare a large number of patients for unnecessarily drug induced site effects.

### Trial status

A total of 227 patients have been screened for *TOP1* expression and we have found 50 patients (22%) to be overexpressing *TOP1* in either primary tumor, a metastatic lesion or both*.*

To date 10 patients have been included.

Initially patients were treated with irinotecan 100 mg/m^2^ but this regimen proved too toxic as two patients withdrew due to severe diarrhoea prior to their first CT evaluation. Accordingly the protocol was amended and following the dose reduction to 75 mg/m^2^ no severe toxicities have been observed.
